# Photobiomodulation (PBM) promotes angiogenesis *in-vitro* and in chick embryo chorioallantoic membrane model

**DOI:** 10.1038/s41598-018-35474-5

**Published:** 2018-11-20

**Authors:** Raimund Winter, Peter Dungel, Frederike Marie Josephine Reischies, Sabrina Rohringer, Paul Slezak, Christian Smolle, Stephan Spendel, Lars-Peter Kamolz, Nassim Ghaffari-Tabrizi-Wizsy, Kurt Schicho

**Affiliations:** 1 Department of Plastic, Aesthetic and Reconstructive Surgery, Medical University of Graz, Graz, Austria; 2 Ludwig Boltzmann Institute for Experimental and Clinical Traumatology, Vienna, Austria; 3 Present Address: Center for Biomedical Sciences, Medical University Vienna, Vienna, Austria; 4 Institute of Pathophysiology and Immunology, SFL Chicken CAM Lab, Medical University of Graz, Graz, Austria; 5 University Clinic for Cranio- Maxillofacial and Oral Surgery, Medical University of Vienna, Vienna, Austria; 6 Research Unit for Safety in Health, Medical University of Graz, Graz, Austria; 7 COREMED–Cooperative Centre for Regenerative Medicine, JOANNEUM RESEARCH Forschungsgesellschaft mbH, Graz, Austria

## Abstract

The application of light in various therapeutic settings known as Photobiomodulation (PBM) is well established. Indications are the improvement of wound healing and tissue regeneration, scarring, and perfusion as well as pain therapy. Tissue perfusion is mandatory for successful wound healing. Nevertheless, there is a lack of mechanistic studies. We investigate the potential effect of PBM from light emitting diodes (LED) at 635 nm, 80 mW/cm^2^, 24 J/cm^2^ on angiogenesis in a two-part study: 1.) Investigation of the effect of PBM on the proliferation of endothelial cells and on vasculogenesis in a co-culture model of endothelial cells and stem cells. 2.) Investigation of the influence of PBM at chick egg chorioallantoic membrane (CAM) assays with fresh human skin xenografts. In both study phases, we observed a stimulating effect of PBM at 635 nm; in part 1: for proliferation of HUVEC (human umbilical vein endothelial cells) (25833 ± 12859 versus 63002 ± 35760 cells/well, p < 0.05, for cellular network formation (2.1 ± 2.1 versus 4.6 ± 3.5, p < 0.05) and for less cell compactness p = 0.01; in part 2: for the increase of number of vessel junctions per ROI (region of interest) (15.9 ± 2.6 versus 20.8 ± 5.4, p < 0.05). Our results suggest significant promotion of angiogenesis by PBM at 635 nm *in vitro* and *in vivo*.

## Introduction

The clinical application of different forms of light is commonly known as Photobiomodulation (PBM). Within the past years, this therapy gained importance in a wide range of medical fields. Recent research typically focuses on the effect of light of different wavelengths, power and energy densities on tissue regeneration, blood perfusion, scarring, anti-inflammatory properties, angiogenesis and pain therapy^1–4^. Although the evidence level of the studies varies to some extent^5^, there is broad consensus in literature that therapeutic use of light can support the treatment of numerous diseases. Regarding the underlying mechanisms of light therapy, several hypotheses are discussed, mainly depending on the distinct properties of the applied light^6,7^. Finding a rationale for light therapy therefore requires further mechanistic and translational studies. Cinically the lack of proper control groups is one of the major difficulties for planning and performing controlled studies due to the fact that e.g. ulcers and wounds can hardly be found in a sufficient number at identical locations, dimensions, with qualitatively and quantitatively same microbial flora in patients, the same patient sex, identical co-morbidities and the same medications. Furthermore, when investigating wound healing, also the genetic predisposition should be considered. Next to classical means of *in-vitro* cell culture and assay analysis methodology, the chick egg chorioallantoic membrane (CAM) assays represents a scientific model that can be categorized as a synthesis between *in-vitro* and *in-vivo* approaches^8^. The chorioallantoic membrane is an extraembryonic membrane of a chick egg. This tissue is vascularized, not innervated and the innate immune system is not fully developed until day 21, which sets ideal conditions for angiogenic and xenografting experiments. CAM assays are not considered animal studies; they are even intended to replace certain animal studies^8^. Considering the fact that increased blood flow and angiogenesis are crucial factors in the course of wound healing, this study aims to investigate a potential stimulating effect of PBM, particularly of LED light of 635 nm at a pulse frequency of 2.5 Hz and a duty cycle of 50%, on angiogenesis. First, the direct effect of light therapy was tested in human endothelial cells, as this cell type is responsible for the formation of new blood vessels. Furthermore, we studied the effect on the formation of tubule-like structures in a co-culture model of human umbilical vein endothelial cells (HUVEC) and adipose-derived stem cells (ASC). Finally, the effects on angiogenesis were investigated in the CAM assay. This is the first controlled study to analyze the effect of LED light of 635 nm at a pulse frequency of 2.5 Hz and a duty cycle of 50% on angiogenesis using CAM.

## Results

### HUVEC proliferation

**Figure 1:**
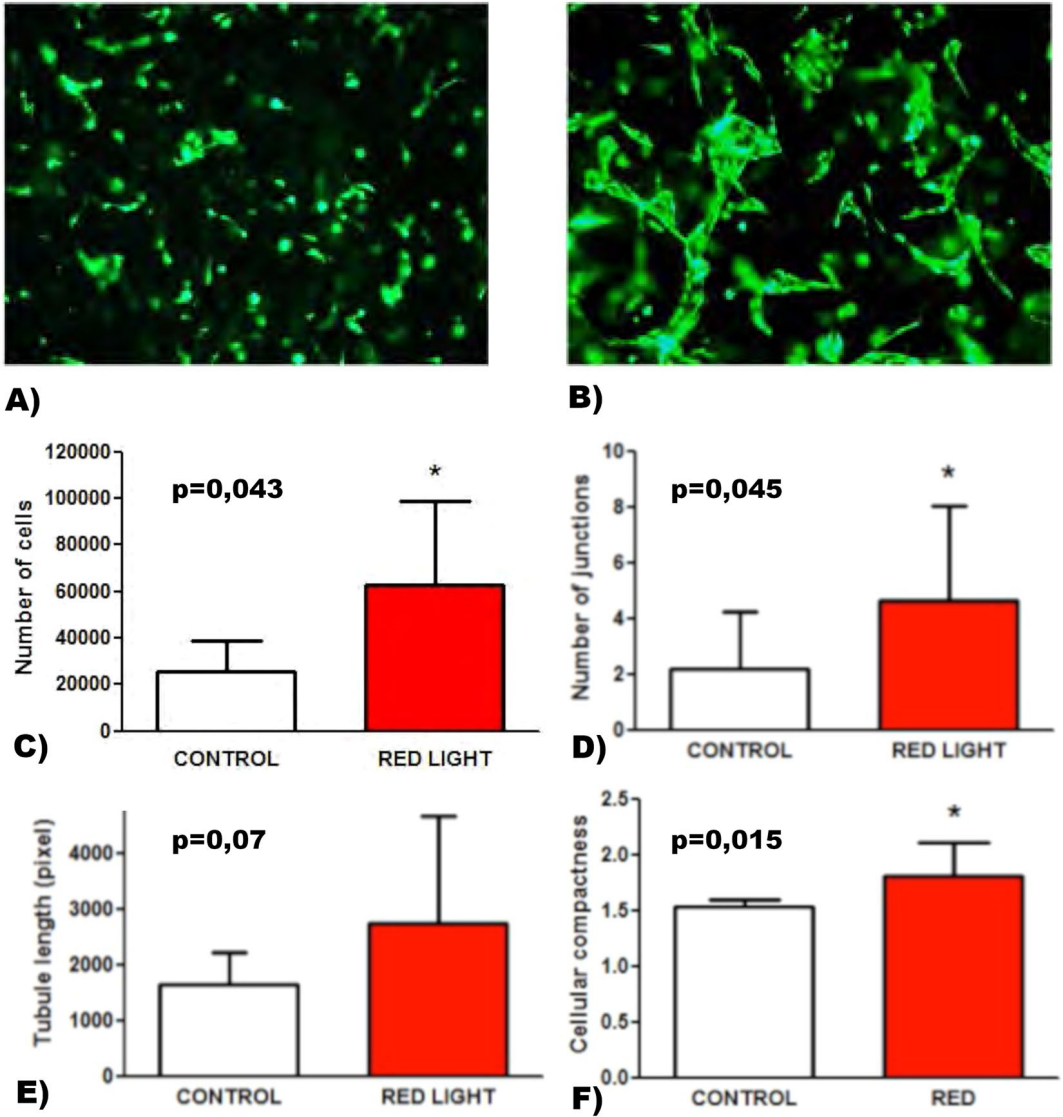
Effects of PBM on 3D vasculogenesis in GFP-HUVEC/ASC co-cultures at a ratio of 1:0.01. Representative images of (**A**) not illuminated control and (**B**) PBM stimulated cells after 7 days. Stimulation of HUVEC with red light significantly increased cell proliferation (**C**), cellular junctions (**D**), tubule length (**E**) and caused significant changes in cell morphology (compactness) (**F**).

The effects of PBM of different wavelengths on proliferation of endothelial cells were evaluated in HUVEC 72 h after light treatment. Stimulation with red light significantly enhanced HUVEC proliferation (63002 ± 35760 per cells per well compared to controls (25833 ± 12859 cells per well) (n = 8, p = 0.043) after 72 h (Fig. 1C).

### Formation of tubular structures *in vitro*

In a 3D co-culture model of GFP-HUVEC and human ASC in a fibrin matrix the PBM treated group displayed a trend towards increased formation of vascular tubes. Figure 1A,B show representative images of stimulated 3D co-cultures after 7 days. Red light positively enhanced network formation of GFP-HUVEC in these co-cultures. The number of junctions were significantly higher in the PBM treated group (Fig. 1D, p < 0.05). Moreover, length of tubules (Fig. 1E, p = 0.07) approached statistically significant increases in the PBM treated group compares to control.

### Compactness

In addition, morphologic changes in the cells could be analyzed. We detected less compactness, which correlates to cell elongation, in the PBM treated group (Fig. 1F, p = 0.01).

### Blood vessels formation in a CAM assay

**Figure 2:**
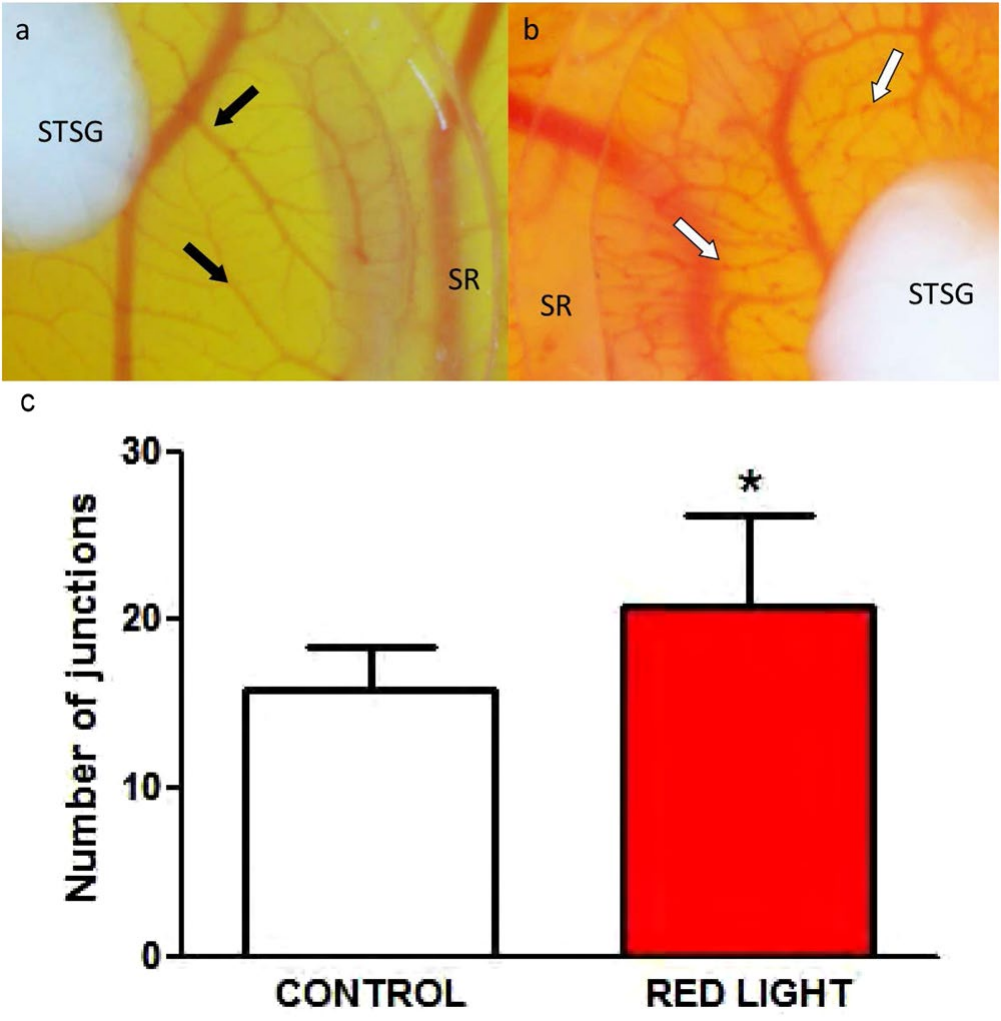
Effects of PBM on angiogenesis in the CAM model. CAMs were treated on four consecutive days for 9 minutes every day with PBM. Effects on angiogenesis were determined by counting the number of junctions in two defined regions of interest, which was significantly (P < 0.05) higher in the group treated with PBM radiation. Control (**a**) and PBM sample (**b**) on day 4 of the experiment. STSG = split thickness skin graft, SR = silicone ring, black arrows = vessels without nodules, white arrows = vessels with multiple nodules.

In order to analyze effects of PBM on angiogenesis in the CAM assays the number of vascular junctions was determined in two rectangular equally sized regions of interest (ROI). The number of branches was significantly higher (20.8 ± 5.4 junctions per ROI), compared to the not illuminated control group (15.9 ± 2.6) (n = 8, p < 0.05) (Fig. 2c).

Investigation of the microscopic images furthermore revealed numerous disseminated nodules (Fig. 2a,b), which can be interpreted as strong indicator of angiogenesis^9,10^.

## Discussion

In the present study we investigated the effects of LED light of 635 nm at a pulse frequency of 2.5 Hz and a duty cycle of 50% on angiogenesis in several models. First, we could confirm the stimulating effect for 2D proliferation of HUVEC and 3D vascular tube formation in fibrin gels. Endothelial cells are vital in wound healing and play a key role in angiogenesis. The stimulation of endothelial cells with light has already been reported [1], however, most of these studies have been performed with lasers. We studied light-induced effects in both 2D as well as 3D experiments with human endothelial cells. 2D proliferation was significantly increased in the light treated group. As endothelial cells are important for angiogenesis we aimed to investigate light-induced network formation in an *in vitro* 3D co-culture model developed in our lab. In this model ASC-derived factors drive endothelial cells to form celluar networks, which are prerequisites for angiogenetic processes. In order to evaluate if light can enhance this network formation, fibrin clots containing HUVEC and ASC at a ratio of 1:0.01 were stimulated with PBM. A seeding ratio of 1:0.01 was chosen in order to observe the stimulating effects of PBM on vasculogenic processes as higher ratios always lead to mature networks. In this model we found a strong trend to increase tube length and a significant increase of number of junctions by light treatment. In addition we also analyzed morphologic changes in the cells (compactness) and detected less compactness, which correlates to cell elongation, in the light treated group. Cell elongation is a necessary prerequisite for network formation and fits into the picture of the enhancement of vasculo- and angiogenesis. The positive effect on endothelial cells was also detected in the chicken CAM bioreactor. We and others believe that the CAM model and its *in-vivo* properties are a meaningful addition and extension of classical *in vitro* methodology^8^.

The mechanism based on which LED light of 635 nm at a pulse frequency of 2.5 Hz and a duty cycle of 50% inflicts its stimulating effects is under discussion. One of the main hypotheses proposes an increase of mitochondrial activity and consequently an enhancement of intracellular adenosine triphosphate (ATP) production in the cells. Furthermore, it was hypothesized that light irradiation causes the release of nitrogen monoxide (NO) leading to enhanced tissue perfusion^11^. Another plausible mechanism is red light as a 12-oxo-leukotriene B4 antagonist^12^, eventually supporting tissue regeneration. Nonetheless, the definitive understanding of the molecular mechanism of stimulating angiogenesis by means of PBM requires further investigations.

We propose that a significant portion of the recognized and well-documented therapeutic effects of light-therapy might be associated with improved blood perfusion, in particular through (neo)angiogenesis; we consequently demonstrated signs of increased micro-perfusion in our models. In a previous study Dungel *et al*. could show that red PBM significantly increased angiogenesis in ischemia-disturbed flap model in rats^13^.

Our findings correspond well with clinical reports in the literature^5^. Nevertheless, clinical experiments in human patients, for instance in ulcer, bear the challenge of designing comparable treatment and control groups, which can be particularly difficult due to multifactorial influences on wound healing, including differences in localization and size of wounds, comparable microbial contamination, co-morbidities and patients’ compliance, as well as demographic and habitual factors such as medication, age, smoking, etc. The CAM assay overcomes this problem as it represents an assay using living embryos under very standardized laboratory conditions.

Pulsed wave mode was used as opposed to continuous light because of its superior stimulating effects at the cellular level^14,15^. There is evidence that pulsing patterns have a more convenient impact on the ability of fibroblasts to produce collagen de novo^16^ and that pulsing patterns have a significantly greater stimulatory effect on cell proliferation and oxidative burst^17^.

A limitation of this study is the fact that comprehensive investigations regarding the variability of vascular properties, e.g. density of blood vessels and capillaries are missing on CAM assays themselves. Further research in this respect is required in order to support controlled studies using this bioreactor in the future. In addition, more sophisticated and automated evaluation methods for the quantification of blood vessels are desirable, for example based on optical coherence tomography and image analysis algorithms.

In conclusion we demonstrated the stimulating effect of intensive LED light of 635 nm at a pulse frequency of 2.5 Hz and a duty cycle of 50% on the angiogenesis *in-vitro* as well as *in-vivo* by means of cell culture and CAM assay. Further research is needed to characterize the mechanistic basis of this observation.

## Methods

All protocols regarding experiments with HUVEC and ASC were approved by the Ethics Committee of the Land Upper Austria. Informed consent was obtained from all subjects (i.e. the stem cell donors). The methods were carried out in accordance with the approved guidelines.

### Cell Culture and Light stimulation

HUVEC were purchased from Lonza (C2519-A or C2856; Lonza, Basel, Switzerland). Green fluorescent protein (GFP) expressing HUVEC were purchased from Olaf pharmaceuticals (GFP; Olaf pharmaceuticals, Worcester, USA). Collection of human adipose tissue was approved by the local ethical board with patients’ informed consent. Subcutaneous adipose tissue was obtained during routine outpatient liposuction procedures under local tumescence anaesthesia. Human ASC were isolated from liposuction material as described before^18^. Cells were cultured in endothelial growth medium (EGM-2; Lonza) supplemented with 5% fetal calf serum (FCS; GE Healthcare, Chalfont St Giles, UK) at 37 °C with 5% CO_2_. Endothelial cells were maintained in cell culture flasks (TPP, Trasadingen, Switzerland) coated with 2 µg/ml bovine fibronectin (Sigma-Aldrich, St. Louis, USA) and ASC were cultured on uncoated plastic surfaces (TPP). For experiments cells were seeded in 24-well plates (Corning Incorporated). Light therapy was applied with Repuls devices (Repuls Lichtmedizintechnik GmbH, Austria). This device emits pulsed LED light of 635 nm (red) at a pulse rate of 50% and a repetition frequency of 2.5 Hz and a peak irradiance intensity of 80 mW/cm^2^ which was determined with a USB 2000 spectrometer (Ocean Optics, FL, USA). Illumination was performed at room temperature for 10 min at a distance of 2 cm with a dose of 24 J/cm^2^. The most important beam parameters^19^ are summarized in Table 1.

**Table 1:** Most important beam parameters^19^.

1. Device Information	
Manufacturer	REPULS Lichtmedizintechnik GmbH Lemböckgasse 61 1230 Wien Austria
Model Identifier	REPULS 7
Year Produced	2017
Number of Emitters	7
Emitter Type	Cree, XPERED-L1-R250-00801
Spatial Distribution of Emitters	130 degrees
Beam Delivery System	Fraen, FCP-N1-XPE1-HRF, 10 degrees
**2. Irradiation Parameters**	
Center wavelength [nm]	632 nm
Spectral bandwidth [nm]	620–635 nm
Operating mode	pulsing
Frequency [Hz]	2.5 Hz
Duty cycle [%]	50%
Energy per pulse [J]	18mJ
Peak radiant power [mW]	140 mW
Average radiant power [mW]	46.6 mW
Aperture diameter [cm]	area/plane sensor with 0.5 cm^2^
Irradiance at aperture [mW/cm^2^]	109 mW/cm^2^
Beam divergence [rad or deg]	10 degress
Beam shape	circular
Beam profile [nm]	10 nm
**3. Treatment Parameters HUVEC**^**a**^/**CAM**^**b**^	
Beam spot size at target [cm^2^]	^a^2.4 cm^2^/^b^ 160 cm^2^
Irradiance at target [mW/cm^2^]	^a^80 mW/cm^2^/^b^ 12 mW/cm^2^
Exposure duration [sec]	^a^10 min/^b^ 9 min
Radiant exposure [J/cm^2^]	^a^24 J/cm^2^/^b^ 3.24 J/cm^2^
Number of points irradiated	1
Area irradiated [cm^2^]	^a^2.4 cm^2^/^b^ 160 cm^2^
Application technique	direct
Total radiant energy (total area) [J]	^a^57.6 J/^b^ 518,4 J

### Proliferation of HUVEC

To assess the effects of light treatment on HUVEC proliferation, 10^4^ HUVEC were seeded to 24-well plates for each condition per time point and grown until 20% confluence. Cells were stimulated by PBM 3 h after seeding and further cultivated for up to 72 hours. Control cells were treated in the same way but were not illuminated. Every day duplicates of cells were enzymatically detached with trypsin (Sigma-Aldrich, St. Louis, MO, USA) and counted with a Neubauer counting chamber (VWR, Darmstadt, Germany).

### HUVEC/ASC co-cultures in 3D fibrin matrices

3D co-cultures of GFP-HUVEC and human ASC in fibrin gels were prepared as described previously^20^. Both cell types were enzymatically detached with trypsin, counted, adjusted to a volume of 95 µl and mixed with 5 µl of 100 mg/ml fibrinogen (Baxter, Vienna, Austria) to a total volume of 100 µl. GFP-HUVEC and ASC were used in a 1:0.01 ratio (10^5^ HUVEC seeded with 10^3^ASC). 3D cultures were treated with light every 24 hours, while control cells were not illuminated. In order to quantify the vascular structure formation in light-stimulated GFP-HUVEC/ASC fibrin matrices, images were taken after 7 days (4 images per clot) on a Leica DMI6000B epifluorescence microscope (Leica, Solms, Germany) in a resolution of 1392 × 1040 pixels and stored in a TIF format. The images were processed with Photoshop CS5 (Adobe Systems, San José, USA) and AngioSys software (TCS Cellworks, London, UK) as described before (Rohringer, Holnthoner *et al*. 2014). Briefly, images were converted to grayscale, the threshold was adapted to eliminate unwanted background, Gaussian blur was applied and single cells were erased manually. Afterwards, the images were loaded to Angiosys software and tubule lengths as well as number of junctions were determined. All experiments were performed 6 times with HUVEC from two different single donors and one pooled donor HUVEC sample to reduce variation in combination with 3 diverging ASC donors for co-culture experiments.

### Compactness

For quantifying changes in cell morphology (compactness) in co-culture at ratios of 1:0.01 after 7 days of incubation, Cellprofiler software version 2.11 was used. Initially a color to gray conversion was performed to obtain gray scale images. Subsequently Cellprofiler’s object detection routine was used to detect the individual cells. No further processing of the images was required. The build in “global background” threshold routine with a threshold correction factor of 2 was used to obtain accurate detection of the cells. Compactness is defined as variance of the radial distance of the objects’ pixels from the centroid divided by the area. Compactness of each cell was measured and correlates with cell elongation. The higher the compactness value, the more elongated cells are. The obtained data was stored in comma separated values format and statistically analyzed.

### CAM assay

This experiment was approved by the Ethical committee at Medical University of Graz (Vote-No: 28-466 ex 15/16). The methods were carried out in accordance with the approved guidelines.

**Figure 3:**
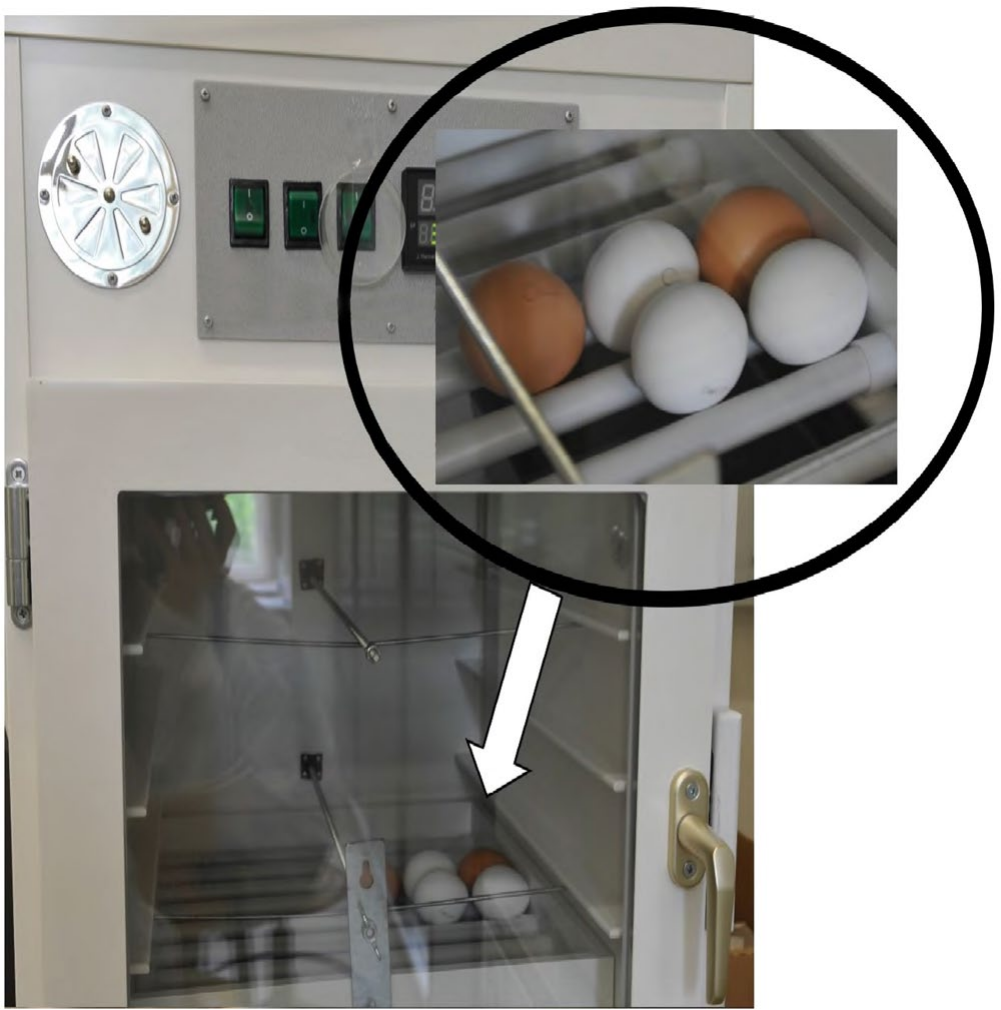
Incubation of fertilized white leghorn chicken eggs at 37 °C.

Fertilized white leghorn chicken eggs were incubated at 37 °C (Fig. 3). On day 3 after incubation, eggs were cracked into sterile plastic shells for ex-ovo cultivation.

### Xenografting

**Figure 4:**
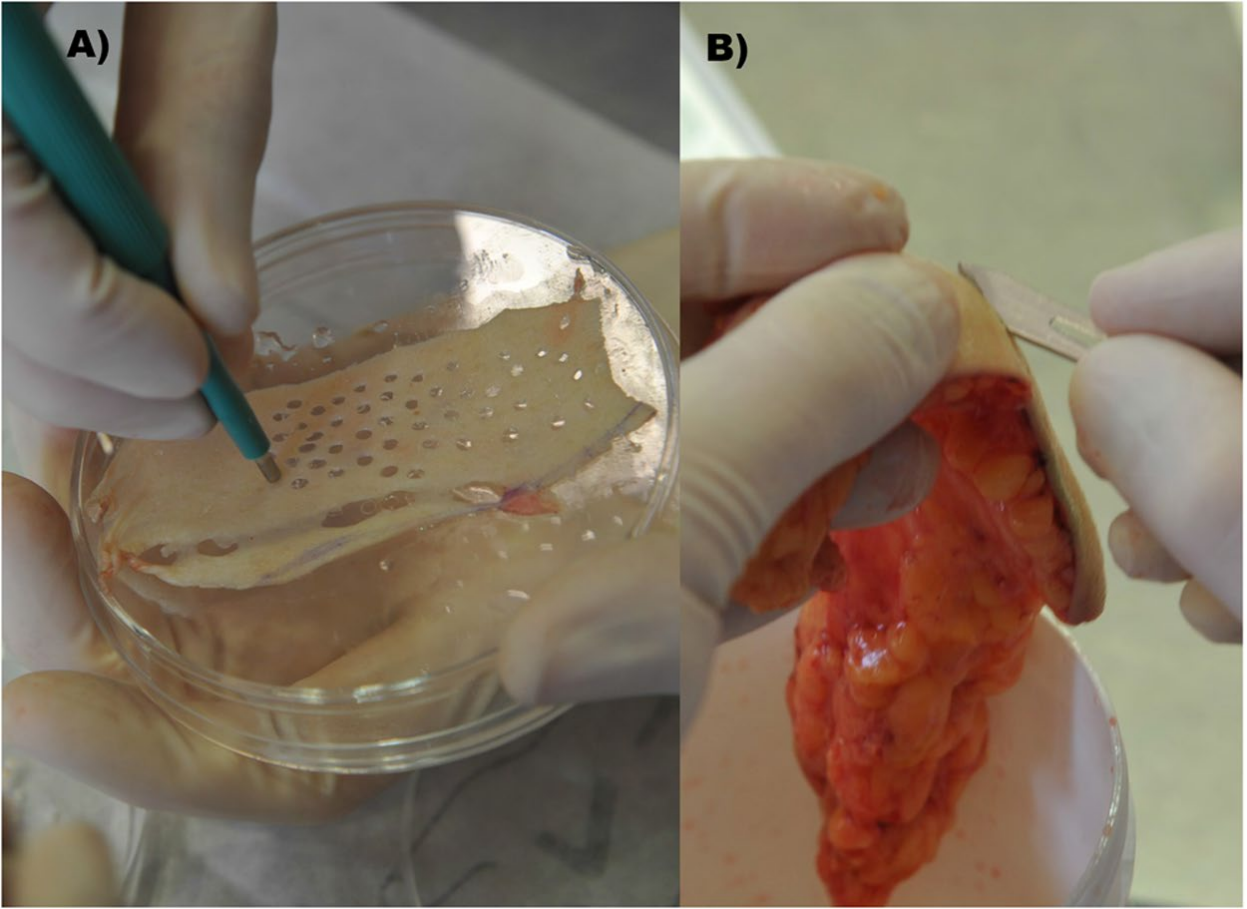
(**A**) Fresh tissue from abdominoplasty surgery. (**B**) Preparation of split thickness skin graft from the surface of the human tissue by means of 5 mm punches.

**Figure 5:**
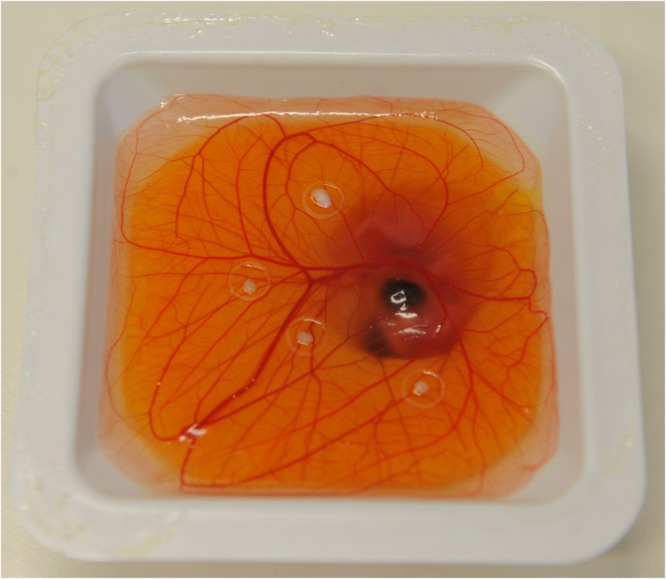
Ex-ovo CAM assay: The four silicone rings at vascular branches, containing the fresh human skin xenografts from abdominoplasty, are cleary perceptible.

**Figure 6:**
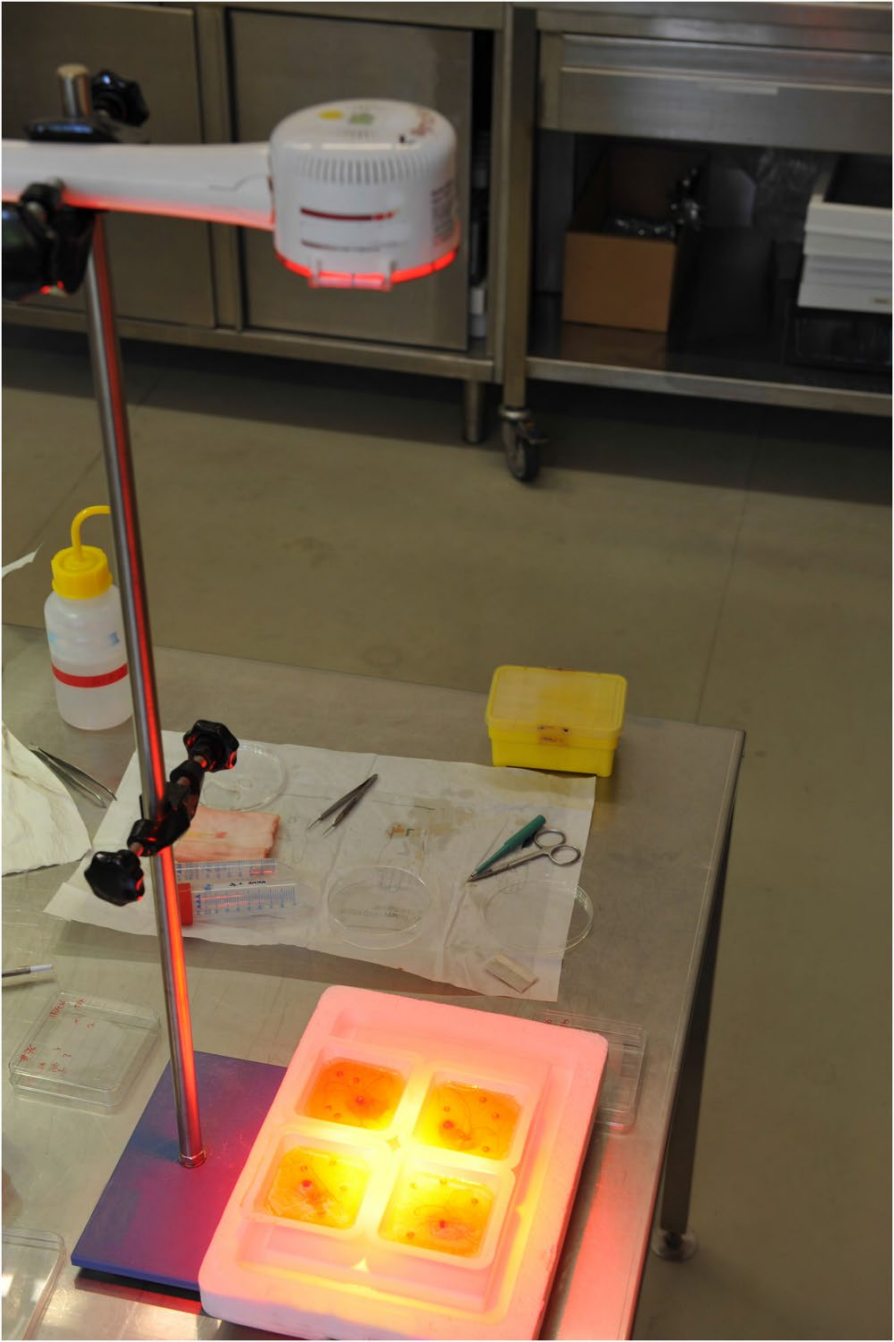
CAM-assays with split thickness skin grafts are incubated at 37 °C for 4 days, only interrupted for the application of LED-light (635 nm, 9 minutes per day) in the treatment-group and microscopic photodocumentation.

**Figure 7:**
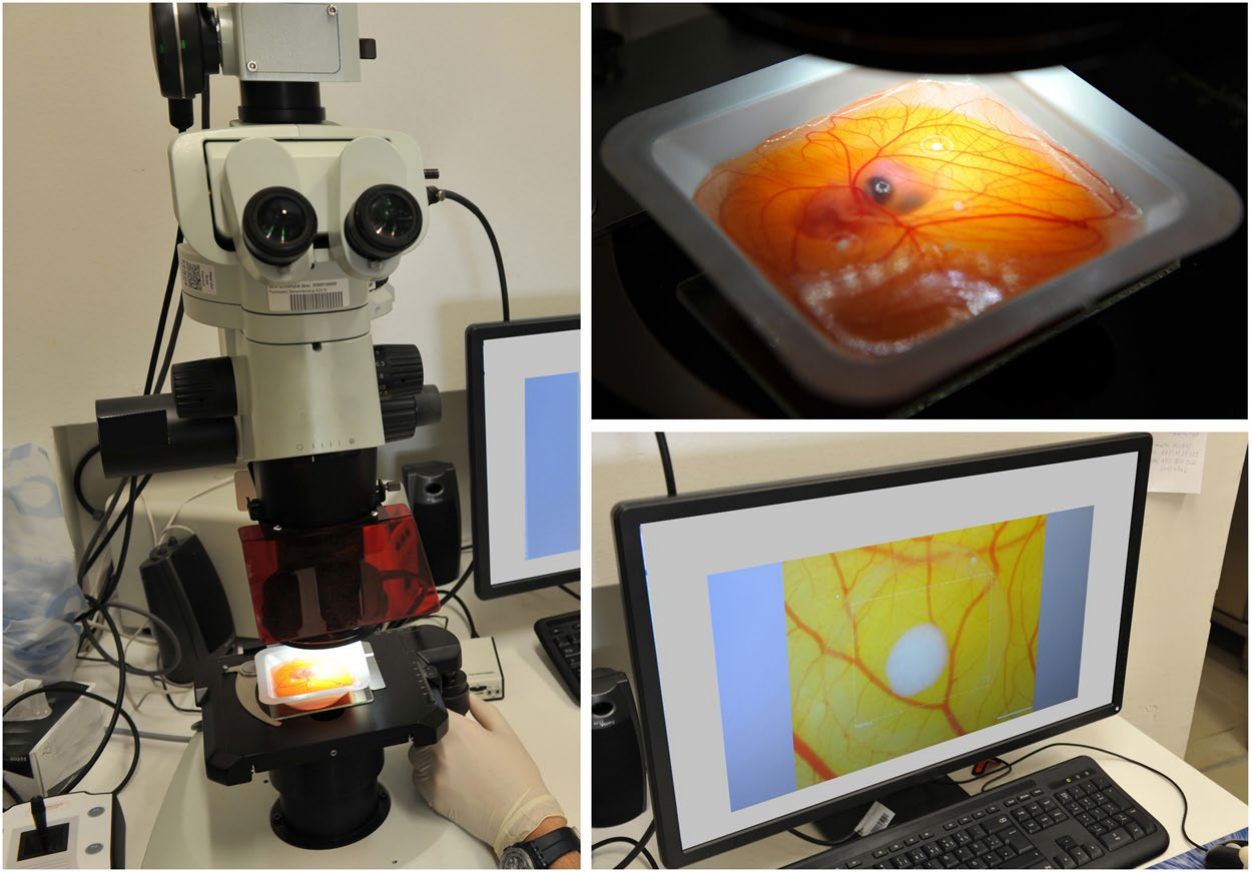
Daily microscopic photodocumentation.

On day 10 chick embryos had reached sufficient size for xenografting and 4 silicone rings with 5 mm diameter were carefully placed on each CAM over vascular branchings. In order to build a proper “bioreactor”, i.e. a model that is suitable for performing “close to *in-vivo*” studies, the xenografting was performed on the same day as an abdominoplasty surgery was conducted at the department of plastic, aesthetic and reconstructive surgery of the University Hospital in Graz. Remnant, to-be-discarded, tissue of this surgical procedures, were transported under sterile conditions from the operating room to the laboratory. Patients had signed informed consent regarding the use of their resected tissue for the purpose of this study according to the legal requirements and regulations of the Biobank at Medical University of Graz. A 0,3 mm Split thickness skin graft was taken from the surface of the human tissue. The split thickness skin graft was punched in circular, 5 mm diameter samples, which were then placed into the silicone rings on the CAM. Four silicone rings with human xenograft skin samples were placed onto each CAM and were further incubated for 3 days to ensure engraftment (Figs 4A,B and 5). As a control group, eight identical CAM-assays were prepared accordingly, each containing four human skin xenografts, i.e. resulting in a total of 16 xenografts in each group. During four consecutive days, in the treatment-group (i.e. 10 CAM-assays), the CAMs were treated with PBM by Repuls7 (Repuls Lichtmedizintechnik GmbH, Vienna) while the control group was not exposed to light. Peak irradiance was 12 mW/cm^2^ which was determined with a USB 2000 spectrometer (Ocean Optics, FL, USA). Illumination was performed at room temperature for 9 min at a distance of 50 cm with a dose of 3.24 J/cm^2^ (Fig. 6). Daily incident light microscopic photo-documentation was carried out in order to achieve the imaging data for consecutive statistical image analysis (Fig. 7). Chick embryos were sacrificed on day 14 by decapitation after anaesthesia by placing them on ice for 5 minutes.

### Medical image analysis

**Figure 8:**
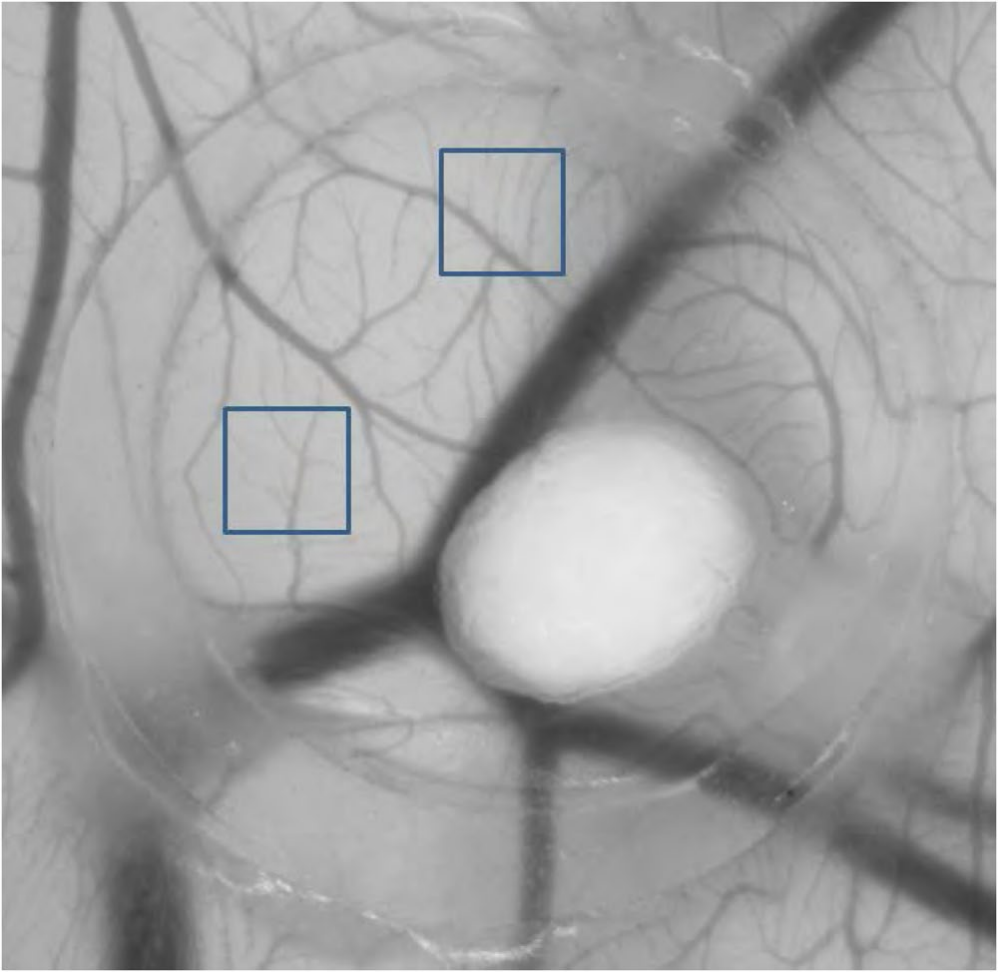
Manual counting of vessel branches in randomized regions of interest.

Randomized pictures of the CAM assays were analysed in a blinded manner. Two rectangular equally sized regions of interest (ROIs) were drawn in each picture and the number of neovascular branches in these ROIs were manually counted by two different investigators (Fig. 8). Each value represents the sum of branches from both ROIs. To maintain comparable analysis parameters throughout the entire dataset, the size of selected ROIs was not changed and used for all images.

### Statistical analysis

Statistical differences were evaluated by two-tailed Student’s t test with prior evaluating the presence of Gaussian distributions. All data sets are presented as mean ± standard deviation. P-values less than 0.05 were considered as significant. All statistical analyses were performed with GraphPad Prism 4.0 software (GraphPad, San Diego, USA). All experiments were performed at least 8 times with HUVEC from two different single donors and one pooled donor HUVEC sample to reduce variation in combination with 3 diverging ASC donors for co-culture experiments.

## Data Availability

he datasets generated during and/or analysed during the current study are available from the corresponding author upon reasonable request.
